# Leptin pro-angiogenic signature in breast cancer is linked to IL-1 signalling

**DOI:** 10.1038/sj.bjc.6606013

**Published:** 2010-12-07

**Authors:** W Zhou, S Guo, R R Gonzalez-Perez

**Affiliations:** 1 Department of Microbiology, Biochemistry & Immunology, Morehouse School of Medicine, Atlanta, GA 30310, USA; 2 Clinic Medicine & Pharmacy College of China Medical University, Shenyang City, Liaoning Province 110002, People's Republic of China

**Keywords:** 4T1 cell, breast cancer, IL-1, leptin, VEGF, VEGFR2

## Abstract

**Background:**

Leptin and interleukin-1 (IL-1) upregulate vascular endothelial growth factor (VEGF), promote angiogenesis and are related to worse prognosis of breast cancer. However, it is unknown whether leptin regulates IL-1, and whether these effects are related to leptin-induction of VEGF/VEGFR2 in breast cancer.

**Methods:**

Several genetic and pharmacological approaches were used to determine the mechanisms involved in leptin regulation of IL-1 system (IL-1*α*, IL-1*β*, IL-1Ra and IL-1R tI) and the impact of IL-1 signalling on leptin-induced VEGF/VEGFR2 expression in mouse mammary cancer 4T1 cells (a model that resembles invasive and highly metastatic human breast cancer).

**Results:**

Leptin increased protein and mRNA levels of all components of the IL-1 system. IL-1 upregulation involved leptin activation of JAK2/STAT3, MAPK/ERK 1/2, PI-3K/AKT1, PKC, p38 and JNK. Leptin-induced phosphorylation of mTOR/4E-BP1 increased IL-1*β* and IL-1Ra expression, but downregulated IL-1*α*. Leptin upregulation of IL-1*α* promoter was linked to SP1 and NF-*κ*B transcription factors. In addition, leptin receptor (Ob-Rb) was upregulated by leptin. Interestingly, leptin upregulation of VEGF/VEGFR2 was partially mediated by IL-1/IL-1R tI signalling.

**Conclusions:**

We show for the first time that leptin induces several signalling pathways to upregulate the translational and transcriptional expression of IL-1 system in breast cancer cells. Moreover, leptin upregulation of VEGF/VEGFR2 was impaired by IL-1 signalling blockade. These data suggest that leptin pro-angiogenic signature in breast cancer is linked to, or regulated, in part by IL-1 signalling.

Obesity, a pandemic in the United States, is associated with more than 100 000 incidents of cancer in the United States every year, particularly cancers of the breast, colon and endometrium. Obese breast cancer patients have increased mortality compared with non-obese (Whiteman *et al*, 2005). Obesity negatively impacts the survival of breast cancer patients regardless of menopausal status, as it has been positively associated with increased risk of recurrence and increased proportion of breast cancer irresponsive to oestrogens (Daling *et al*, 2001). The obese gene ligand (leptin), a 16-kDa cytokine, is mainly produced by adipose tissue. Higher levels of leptin are found in female, postmenopausal women and obese individuals. Leptin is a pro-angiogenic, pro-inflammatory and mitogenic factor, the actions of which are reinforced through crosstalk with cytokines/growth factors (Caldefie-Chézet *et al*, 2005; Gonzalez *et al*, 2006; Rene Gonzalez *et al*, 2009; Jarde *et al*, 2010).

Breast carcinoma cells express higher levels of leptin and its receptor, Ob-R, than normal mammary cells (Miyoshi *et al*, 2006), and a significant correlation between leptin/Ob-R levels with metastasis and lower survival of breast cancer patients has been found. Moreover, studies on leptin (*ob/ob*) and Ob-R (*db/db*) mutant mice have provided compelling data supporting a role for leptin in breast cancer development. These obese mice with deficiency in leptin signalling show a significantly lower incidence of mammary tumours than their lean littermates. MMTV/TGF-*α* mice have a proclivity to develop mammary tumours, but when crossed with leptin/Ob-R-deficient mice, there is a reduced incidence of mammary tumours in their progeny (Cleary *et al*, 2003, 2004). Furthermore, our published data strongly suggest that leptin is an important factor for breast cancer development. We have shown that the inhibition of leptin signalling *in vitro* and *in vivo* by our innovative leptin peptide receptor antagonists (PEG-LPrA) significantly decreased the levels of vascular endothelial growth factor (VEGF) and its receptor type 2 (VEGFR2) before hypoxia is manifested (Gonzalez-Perez *et al*, 2010) in breast cancer and stroma cells, while simultaneously reducing establishment and growth of tumours in syngeneic (Gonzalez *et al*, 2006), xenograft (Rene Gonzalez *et al*, 2009) and 7,12-dimethylbenz(a)anthracene-diet-induced obesity (unpublished) mouse models of breast cancer.

One critical event for tumour growth and metastasis success is the growth of a new network of blood vessels that can be promoted by several cytokines derived from immune, endothelial, epithelial and stromal cells. Leukaemia inhibitory factor, VEGF, interleukin-1 (IL-1) and leptin are known angiogenic factors expressed by mammary cancer cells (Apte *et al*, 2006a, 2006b). Aberrant inflammatory response over normal immunity facilitates the development of neoplasias. Increased levels of IL-1 are found in breast cancer (Miller *et al*, 2000). IL-1 family, one of the major pro-inflammatory cytokines, is represented by two ligands: IL-1*α* and IL-1*β*, an antagonist: IL-1 receptor antagonist (IL-1Ra) and two receptors: IL-1R tI (type I receptor) and IL-1R tII (type II receptor) (Boraschi *et al*, 1996). IL-1R tI is an 80-kDa protein, which has an intra-cytoplasmic domain of about 215 amino acids. IL-1R tI, is the only receptor responsible for transmitting signalling upon IL-1 binding. In contrast, IL-1R tII, a 60-kDa protein with a short intra-cytoplasmic domain (29 amino acids) serves as a decoy target that reduces the levels of IL-1. IL-1Ra is an inhibitor protein that can bind to IL-1R tI or IL-1R tII with similar affinity without triggering cell signalling effects. Therefore, IL-1Ra represents a natural inhibitor of IL-1 signalling (Apte *et al*, 2006a, 2006b).

IL-1 is a known inducer of VEGF expression in different tissues and has been described as a factor in cancer development (Carmi *et al*, 2009; Valdivia-Silva *et al*, 2009). Macrophages are recruited to tumours by chemokines, cytokines and growth factors, including VEGF, produced by tumour cells and other cell types in the tumour microenvironment. In turn macrophages and tumour cells secrete IL-1 that contributes to tumour progression by facilitating angiogenesis, matrix remodelling, invasion and metastasis (Chen *et al*, 2009). Strikingly, the inhibition of IL-1 signalling by exogenous IL-1Ra negatively impacted tumour angiogenesis in nude mice (Voronov *et al*, 2003).

Although, all the mechanism(s) by which leptin contributes to tumour progression are unknown, our published data suggest that specific leptin signalling increase cancer-cell proliferation and the expression of VEGF/VEGFR2 in breast (Gonzalez *et al*, 2006; Rene Gonzalez *et al*, 2009; Gonzalez-Perez *et al*, 2010; Guo *et al*, 2010) and endometrial cancer (Carino *et al*, 2008), and in endometriotic lesions (Styer *et al*, 2008). The associated expression of leptin and IL-1 has been described in several pathological situations. In endometrial cancer cells leptin induces the expression of IL-1 system (Carino *et al*, 2008). Leptin and IL-1 signalling can activate NF-*κ*B and increase the levels of VEGF and bcl-2 that could be linked to breast cancer progression (Caldefie-Chézet *et al*, 2005). Therefore, leptin and IL-1 may have synergistic functions in breast cancer progression. Moreover, their relationships might be more evident in obese (showing higher leptin levels) than in lean individuals.

Despite that both leptin (Caldefie-Chézet *et al*, 2005; Gonzalez *et al*, 2006; Rene Gonzalez *et al*, 2009; Gonzalez-Perez *et al*, 2010; Guo *et al*, 2010) and IL-1 systems (Kumar *et al*, 2003; Voronov *et al*, 2003) seem to have important roles in tumour angiogenesis and growth no published data is available on the potential relationships between leptin and IL-1 signalling in breast cancer. We hypothesise that the leptin-induced progression of breast cancer could involve the regulation of IL-1 system expression and activation of NF-*κ*B and/or SP1. Moreover, leptin-induced IL-1 could be related to leptin upregulation of essential pro-angiogenic factors in breast cancer: VEGF/VEGFR2. To test this hypothesis inhibitors of leptin-induced kinases, RNA knockdown for transcription factors and luciferase reporter for IL-1 gene promoter were used in mouse mammary 4T1 cells, a model that closely resembles human invasive/metastasic stage IV breast cancer (Gonzalez *et al*, 2006; Gonzalez-Perez *et al*, 2010). Present data suggest that leptin upregulates the IL-1 system at the transcriptional and translational levels. Remarkably, leptin-induced increase in VEGF and VEGFR2 levels were abrogated by the blockade of IL-1 signalling. Therefore, leptin pro-angiogenic actions in breast cancer may be linked to, or regulated in part by IL-1 signalling.

## Materials and methods

### Reagents and antibodies

Recombinant mouse leptin and mouse IL-1*β*, IL-1*α*, IL-1Ra and VEGF ELISA Kits were from R&D Systems (Minneapolis, MN, USA). Fetal bovine serum was obtained from Gemini Bioproducts (West Sacramento, CA, USA), RPMI-1640 medium and penicillin–streptomycin cocktails were from American Type Culture Collection (Manassas, VA, USA). Antibodies for IL-1R tI, Ob-R-NH2, Ob-Rb-COOH, goat IgG2b, SP1 small interference (si) RNA and control siRNA-A were obtained from Santa Cruz Biotechnology, Inc. (Santa Cruz, CA, USA). Antibodies for IL-1*α*, IL-1*β*, mTOR and *β*-actin were from Abcam Inc. (Cambridge, MA, USA). Antibodies for pERK 1/2/pMAPK (Thr202/Ty204), pmTOR (S2448), p4E-BP1 (Ser65) and p70S6 Kinase-1 (Ser371) were from Cell Signalling (Danvers, MA, USA). Anti-phospho AKT1/PKB*α* (Ser473) was from Upstate (Lake Placid, NY, USA). Horseradish peroxidase conjugates, iScript cDNA Synthesis, IQ SYBR Green Supermix and protein determination kits were from Bio-Rad Laboratories (Hercules, CA, USA). The ECL western blot stripping buffer was from Thermo Scientific (Rockford, IL, USA). Dual-luciferase assay system and control pGL-3 plasmid were obtained from Promega (Madison, WI, USA). The NF-*κ*B1 mouse short-hairpin RNA was from Origene (Rockville, MD, USA). Nuclear extract kit was from Active Motif (Carlsbad, CA, USA). The RNeasy and DNase kits and Superfect transfect reagents were obtained from Qiagen (Valencia, CA, USA). Wortmannin, AG490, PD98059, Gö6976, SB203580, SP600125, Rapamycin, RIPA buffer, Endofree plasmid maxiprep kit, protease inhibitor and phosphatase inhibitor cocktails and other chemicals were from Sigma-Aldrich (St Louis, MO, USA).

### Cell culture

The mouse mammary tumour cell line 4T1 (CRL-2539; American Type Culture Collection) was cultured on uncoated flat-bottomed plastic plates (cell densities of 1.0, 2.0 or 4.0 × 10^5^ cells per well for 24-, 12- or 6-well plates as described elsewhere (Gonzalez-Perez *et al*, 2010). Semi-confluent cells were starved for 24 h in basal medium (RPMI-1640 without fetal bovine serum) and treated with different compounds. In all experiments triplicate wells, tubes and reactions were run and repeated at least three times with different cell preparations.

### Leptin dose–response and time–course effects

4T1 cells were starved as described above and incubated for 24 h with medium containing leptin (0, 0.6, 1.2 and 6.25 nM, equivalent to 0, 10, 20 and 100 ng ml^−1^). Cell lysates and culture supernatants were used to assess leptin dose–response effects on the levels of IL-1 system component proteins and mRNAs as determine by ELISA, western blot and real-time RT–PCR, respectively. For leptin time–course effect analyses, 4T1 cells were treated with 1.2 nM leptin for 0, 4, 8, 12, 24 and 48 h. Protein concentrations in cell lysates were determined by the Bradford method (Bio-Rad Laboratories) and levels of IL-1R tI were assessed by western blot.

### Specific kinases involved in leptin-mediated effects on IL-1 system

4T1 cells were incubated with 1.2 nM leptin and kinase inhibitors (AG490 for JAK2/STAT3, 30 *μ*M; PD98059 for MEK/MAPK/ERK1/2, 30 *μ*M; wortmannin for PI-3K/AKT1, 20 *μ*M; Gö6976 for PKC-Ca dependant, 30 *μ*M; SB203580 for p38 kinase, 30 *μ*M; SP600125 for JNK, 30 *μ*M; and Rapamycin 20 *μ*M for mTOR) for 24 h. Protein levels of IL-1 system components in supernatants and cell lysates were determined by ELISA and western blot, respectively.

### Reporter gene transfection and luciferase assay

Semi-confluent 4T1 cells were transiently cotransfected with 50 ng of a *Renilla* reporter-luciferase control plasmid and 500 ng of pGL3-IL-1*α* plasmid (kindly provided by Dr Eugenie S Kleinerman and Dr Ying Cao, University of Texas MD Anderson Cancer Center). After 3 h of cotransfection, cells were incubated with 1.2 nM leptin for 24 h and luciferase activity was determined. Normalization was based on cotransfected *Renilla* luciferase activities.

### RNA extraction and real-time RT–PCR

RNA was extracted from 4T1 cells and first-strand cDNA was synthesised using SuperScript II reverse transcriptase. The cDNA was used as a template in real-time RT–PCR reactions, as described elsewhere (Gonzalez-Perez *et al*, 2010). For generating a standard curve, amplified cDNA was used in a five-fold dilution series of 100–0.16 ng cDNA per reaction. Relative gene expression was calculated using the glyceraldehyde-3-phosphate dehydrogenase (GAPDH) expression value. Primers used in the experiment were as following, mouse IL-1*α* forward: 5′-TCGGGAGGAGACGACTCTAA-3′ and reverse: 5′-AGGTCGGTCTCACTACCTGTG-3′ mouse IL-1*β* forward: 5′-TGCACTACAGGCTCCGAGAT-3′ and reverse: 5′-CGTTGCTTGGTTCTCCTTGT-3′ mouse IL-1Ra forward: 5′-TGTGTTCTTGGGCATCCAC-3′ and reverse 5′-TTCTCAGAGCGGATGAAGGT-3; mouse IL-1R tI forward: 5′-GTCTTGGAGGGACAGTTTGG-3′ and reverse: 5′-CAGCTGAAGCCTCCCATATC-3′ mouse VEGFR2 forward: 5′-GTGATTGCCATGTTCTTCTGGC-3′ and reverse: 5′-TTCATCTGGATCCATGACAA-3′ mouse VEGF forward: 5′-TACCTCCACCATGCCAAGTGGT-3′ and reverse: 5′-AGGACGGCTTGAAGATGTAC-3′. The GAPDH was used as internal control using the following primers: forward: 5′-TGCACCACCAATGCTTAG-3′ and reverse: 5′-GGATGCAGGGATGATGTTC-3′.

### Western blot analysis

Following cytokine and antibody treatment, cellular lysates were prepared for western blot as described elsewhere (Gonzalez-Perez *et al*, 2010). *β*-actin was used as control.

### Flow cytometry assay

4T1 cells were incubated in basal medium for 24 h (to synchronise cells) and further incubated with 1.2 nM leptin for additional 24 h. Cells were re-dispersed, permeabilized and incubated with antibodies for NH2 (all isoforms) and C-terminal (long-isoform) Ob-R domains as described elsewhere (Gonzalez *et al*, 2003). Goat anti-*β*-actin antibodies were used as positive control. Cells were analysed by fluorescence-activated cell sorting (BD FACScan, Becton Dickinson, Franklin Lakes, NJ, USA) and, data were analyzed using BD FACSDiva (Becton Dickinson) and FlowJo (TreeStar, Ashland, OR, USA) software.

### Immunocytochemistry

To assess leptin effects on IL-1 system expression 4T1 cells (5 × 10^5^ cells per chamber) were cultured in immunocytochemistry-treated glass slides (BD Falcon, Belford, MA, USA) and incubated with 1.2 nM leptin and antibodies for IL-1*α*, IL-1*β* and IL-1R tI (Johnston *et al*, 2008). Negative controls have omitted the primary antibody.

### RNA knockdown

Semi-confluent 4T1 cells were cotransfected with SP1 siRNA oligonucleotide, NF-*κ*B1 shRNA and pGL3-IL-1*α* plasmids, and treated with 1.2 nM leptin for 24 h. Luciferase activities were determined as described above.

### Blockade of IL-1R tI

4T1 cells were incubated with 1.2 nM leptin and anti-mouse IL-1R tI antibody or non-specific species-matched IgG2b (0.1 *μ*g ml^−1^). mRNA levels and VEGF protein were determined by ELISA and real-time RT–PCR, respectively. The VEGF and VEGFR2 proteins in cell lysates were analyzed by western blot.

### Data analysis

Student's *t*-test was used for data analysis. Data are presented as mean±s.e.m. Values for *P*<0.05 were considered statistically significant. The model included the main effects of treatments and replicates.

## Results

### Leptin dose – response induction of IL-1 system

**Figure 1:**
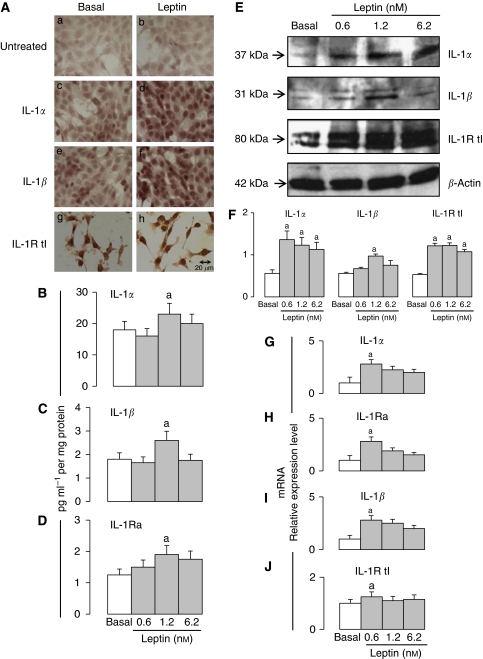
Leptin induces the expression of IL-1 system in 4T1 cells. (**A**) Representative results of leptin-induced increase in protein levels of IL-1 system as determined by immunocytochemistry (magnification × 40). Control cells in basal conditions: (Aa) no antibodies; (Ac) IL-1*α* antibodies; (Ae) IL-1*β* antibodies; (Ag) IL-1R tI antibodies. Cells incubated with leptin: (Ab) no antibodies; (Ad): IL-1*α* antibodies; (Af) IL-1*β* antibodies and (Ah) IL-1R tI antibodies. Protein levels of IL-1 ligands (**B**, IL-1*α*; **C**, IL-1*β*) and antagonist (**D**, IL-1Ra) as determined by ELISA (pg ml^−1^ per mg protein). (**E**), Protein levels of IL-1 ligands and receptor as determined by western blot (WB). (**F**) WB results were normalised to *β*-actin as a control and densitometric analysis of bands was carried out with the imageJ software. mRNA levels of IL-1 ligands (**G**, IL-1*α*; **I**, IL-1*β*), antagonist (**H**, IL-1Ra) and receptor (**J**, IL-1R tI) as determined by real-time RT–PCR. GAPDH was used as internal control. 4T1 cells were cultured for 24 h and leptin dose-induced (0, 0.6, 1.2 and 6.2 nM) effects were determined as described (see Materials and Methods). (a) *P*<0.05 when comparing levels of protein or mRNA to control (basal). Data (mean±s.e.) are representative of the results derived from a minimum of three independent experiments.

Leptin induced the expression of all IL-1 system components at protein and mRNA levels (Figure 1). Leptin-mediated increase in IL-1 protein levels was initially determined by immunohistochemistry (Figure 1A). Leptin (1.2 nM) increased protein levels of IL-1*α*, IL-1*β* and IL-1Ra as determined by ELISA. Leptin effects show bell-shaped dose–response patterns. Leptin upregulation of IL-1 protein levels were found significant at 1.2 nM (Figure 1B–D). Moreover, leptin upregulation of IL-1 mRNA showed significant changes at lower concentration, that is, 0.6 nM (Figure 1G–J). Western blot analysis showed that leptin at all doses tested increased the levels of IL-1*α*, IL-1*β* and IL-1R tI (Figure 1E and F).

### Leptin time–course regulation of IL-1 system

**Figure 2:**
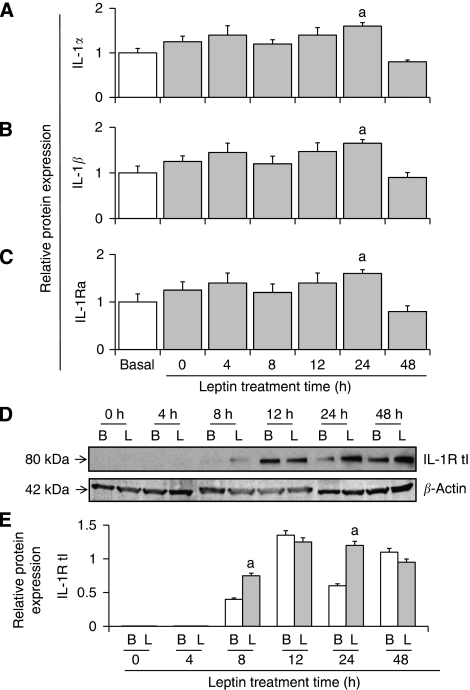
Time–course response for leptin-mediated effects on the expression of IL-1 system in 4T1 cells. Leptin effects on protein levels of IL-1*α* (**A**), IL-1*β* (**B**), IL-1Ra (**C**) as determined by ELISA. Levels of cytokines were normalised to basal condition (assigned as 1) and expressed in arbitrary units. IL-1R tI (**D**) as determined by western blot (WB). (**E**) WB results for IL-1R tI were normalised to *β*-actin as a control and densitometric analysis of bands was carried out with the imageJ software. 4T1 cells were cultured in a medium containing 1.2 nM leptin for 0–48 h. (a) *P*<0.05 when comparing levels of protein to control (basal). Data (mean±s.e.) are representative of the results derived from a minimum of three independent experiments.

Protein levels of all components of IL-1 system in 4T1 cells were increased after 24 h of incubation with leptin (Figure 2A–E).

### Leptin signalling pathways involved in the regulation of IL-1 system

**Figure 3:**
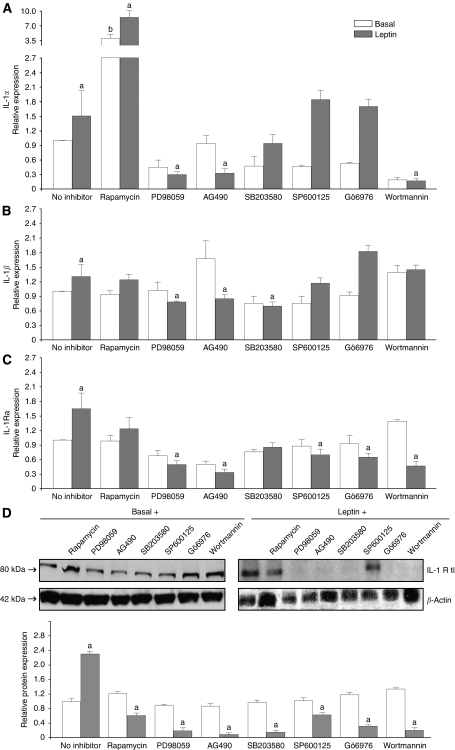
Leptin-induced signalling pathways involved in the regulation of IL-1 system in 4T1 cells. Effects of leptin and kinase inhibitors on levels of IL-1*α* (**A**), IL-1*β* (**B**), IL-1Ra (**C**) and IL-1R tI (**D**) as determined by ELISA and western blot, respectively. 4T1 cells were treated with leptin (0 or 1.2 nM) for 24 h in the presence of inhibitors of JAK2/STAT3 (AG490, 30 *μ*M), MEK/MAPK/ERK1/2 (PD98059, 30 *μ*M), PI-3K/AKT1 (wortmannin, 20 *μ*M), PKC-Ca dependant (Gö6976, 30 *μ*M), p38 kinase (SB203580, 30 *μ*M), JNK (SP600125, 30 *μ*M) and mTOR (Rapamycin, 20 *μ*M) signalling pathways. WB results were normalised to *β*-actin as a control and densitometric analysis of bands was carried out with the imageJ software. (a) *P*<0.05 and (b) *P*<0.01 when comparing levels of protein to control with or without inhibitors (basal), respectively. Data (mean±s.e.) are representative of the results derived from a minimum of three independent experiments.

Leptin increased the levels of IL-1*α* (Figure 3A), IL-1*β* (Figure 3B) and IL-1Ra (Figure 3C). These effects were related to leptin-induced canonical signalling pathways (JAK2/STAT3, MAPK and PI-3K/AKT1). Meanwhile, leptin induction of IL-1*β* (Figure 3B) and IL-1Ra levels (Figure 3C) was also related to mTOR activation. However, mTOR seems to negatively regulate IL-1*α* (Figure 3A). Furthermore, leptin regulation of IL-1*β* (Figure 3B) and IL-1Ra (Figure 3C) to some extent involved JNK and PKC and p38 kinases. In contrast, several leptin signalling pathways were involved in the increase of IL-1R tI levels (Figure 3D).

**Figure 4:**
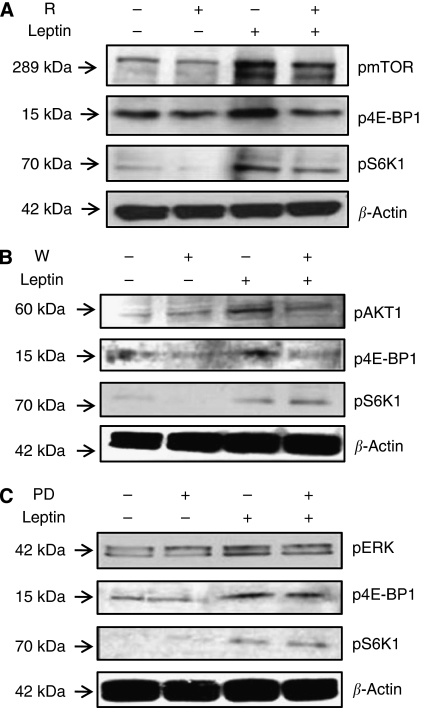
Leptin induces the phosphorylation of mTOR, 4E-BP1 and p70S6K1 in 4T1 cells. Representative western blot results for leptin-induced activation of mTOR and its downstream targets (4E-BP1 and S6K1) after treatment of 4T1 with kinase inhibitors: (**A**) rapamycin, (R for mTOR); (**B**) wortmannin (W, for PI-3K/AKT1) and (**C**) PD98059 (PD for MAPK). 4T1 cells were treated with leptin (0 or 1.2 nM) and inhibitors for 24 h.

We further assessed that leptin regulation of IL-1 system involved the phosphorylation of mTOR downstream targets: 4E-BP1 and 70S6K1 (Figure 4). These leptin effects were abrogated by rapamycin (Figure 4A). On the other hand, wortmannin abrogated AKT1 and 4E-BP1 phosphorylation, but no effects on p70S6K1 were found (Figure 4B). In contrast, inhibition of pERK 1/2 did not affect p4E-BP1 or p70S6K1 levels (Figure 4C).

### Leptin effects on the regulation of IL-1*α* promoter

**Figure 5:**
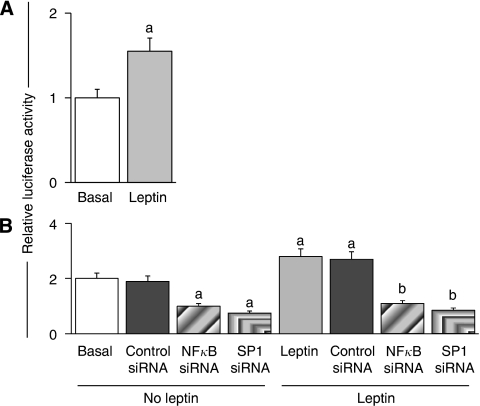
Leptin induces expression of IL-1*α* gene. (**A**) leptin transcriptional activation of IL-1*α* reporter, (**B**) effects of siRNA for SP1 and NF-*κ*B. 4T1 cells were transiently transfected with a IL-1*α* reporter construct and treated with leptin (0 and 1.2 nM) and siRNA-SP1 and shRNA–NF-*κ*B for 24 h. Luciferase activity was determined as described (see Materials and Methods) and expressed as a percent of basal or leptin-treated cells. (a) and (b) *P*<0.05 when comparing levels of luciferase activity to control (basal) or leptin-treated cells, respectively. Data (mean±s.e.) are representative of the results derived from a minimum of three independent experiments.

Incubation of cells with leptin increased more than 50% the activity of IL-1*α* promoter compared with control (*P*<0.05) (Figure 5A). The RNA silencing of SP1 and NF-*κ*B negatively affected leptin-mediated induction of IL-1*α* reporter activity (Figure 5B).

### Leptin upregulates its receptor in 4T1 cells

**Figure 6:**
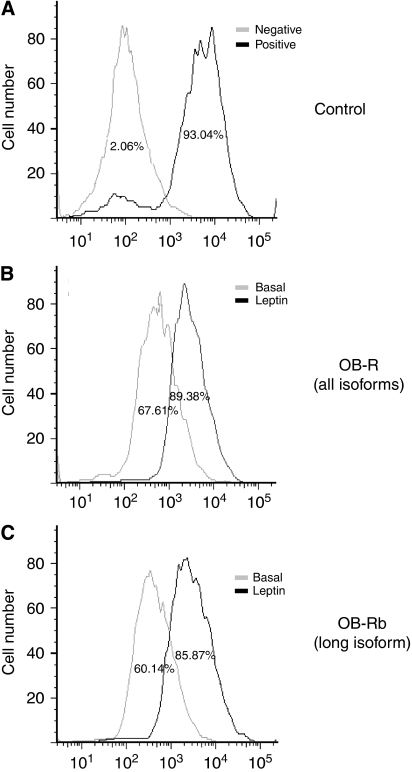
Leptin upregulated OB-R in 4T1 cells. Representative histograms from flow cytometric determination of leptin effects on the expression of OB-R in 4T1 cells. (**A**) Positive control (*β*-actin antibody) and negative control (isotype-matched unspecific IgG), (**B**) basal and leptin-induced expression of OB-R all isoforms (anti-OB-R NH2-terminus antibody), (**C**) basal and leptin-induced expression of OB-Rb (long isoform; anti-OB-Rb COOH-terminus antibody). 4T1 cells were cultured in medium containing 1.2 nM leptin for 24 h, incubated with specific antibodies and analyzed by flow cytometry. Data derived from a minimum of three independent experiments were analysed using BD FACSDiva and FlowJo software.

4T1 cells express Ob-R in basal conditions. Leptin induced a similar increase of Ob-R total (22% Figure 6B) and Ob-Rb (26% Figure 6C). These results suggest that leptin mainly increases the expression of the full-functional Ob-Rb.

### Impact of IL-1R tI blockade on leptin-induction of VEGF/VEGFR2

**Figure 7:**
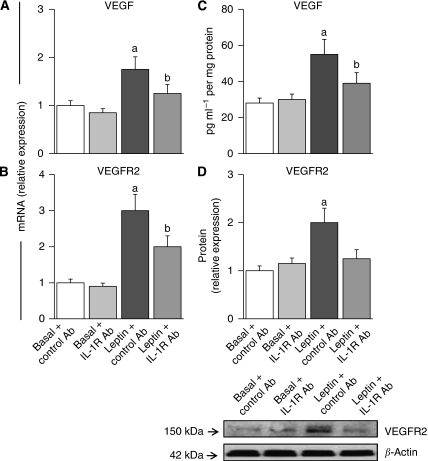
The blockade of IL-1R tI negatively impacts on leptin upregulation of VEGF/VEGFR2 expression in 4T1 cells. Effects of leptin and IL-1R tI-blocking antibodies on levels of VEGF (**A**, mRNA and **C**, protein) and VEGFR2 (**B**, mRNA and **D**, protein) in 4T1 cells. Cells were incubated with leptin (0, 1.2 nM) and anti mouse IL-1R tI antibody (0.1 *μ*g ml^−1^) for 24 h. Cells incubated with non-specific species-matched IgG2b served as negative controls. The VEGF and VEGFR2 mRNA levels were quantified by real-time RT–PCR and normalised to the glyceraldehyde-3-phosphatase dehydrogenase expression. The VEGF and VEGFR2 protein were determined by ELISA and western blot (WB), respectively. The VEGFR2 results from WB were analysed by densitometric analysis (imageJ software) and normalised to *β*-actin as a control. (a) *P*<0.01 when comparing basal to leptin+control Ab and (b) *P*<0.05 when comparing leptin+IL-1R tI to leptin+control Ab. Data (mean±s.e.) are representative of the results derived from a minimum of three independent experiments.

Leptin induced almost two-fold and three-fold increase in the levels of VEGF mRNA (Figure 7A) and protein (Figure 7C), respectively. Similarly, leptin induced three-fold and two-fold VEGFR2 mRNA (Figure 7B) and protein (Figure 7D), respectively. The addition of IgG control antibodies did not alter leptin-mediated effects on VEGF (Figure 7A and C) or VEGFR2 (Figure 7B and D). The blockade of IL-1R tI function significantly impaired leptin's effects on VEGF and VEGFR2 (see Figure 7 A–D).

## Discussion

**Figure 8:**
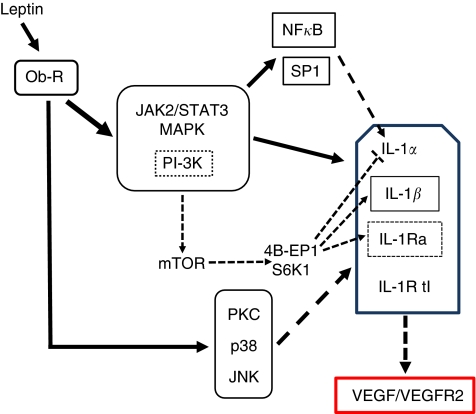
Signalling mechanisms for leptin regulation of IL-1 system in 4T1 cells. Leptin upregulated IL-1 system at transcriptional and translational levels. Leptin canonical signalling pathways (JAK2/STAT3, MAPK and PI-3K/AKT1) were mainly involved in the upregulation of IL-1 system. Leptin activation of PI-3K/AKT1 was related to the phosphorylation of mTOR and its downstream target, 4E-BP1, which is probably linked to upregulation of IL-1*β* and IL-1Ra. In addition, mTOR negatively regulated IL-1*α*. Leptin upregulation of IL-1*α* promoter involved the activation of SP1 and NF-*κ*B. Leptin-induced non-canonical signalling pathways (PKC, p38 and JNK) differentially impacted on the expression of some components of IL-1 system. Leptin-induced upregulation of VEGF/VEGFR2 system was partially mediated by IL-1/IL-1R tI signalling.

Leptin regulates inflammatory cytokines, including IL-1, in diverse tissues and pathological conditions (Chala *et al*, 2006; Carino *et al*, 2008; Johnston *et al*, 2008). Nevertheless, published studies on the relationships between leptin and IL-1 in breast cancer are scarce (Miller *et al*, 2000). To address whether leptin could regulate IL-1 system in breast cancer, a mouse mammary cancer cell line 4T1 that closely resembles human invasive breast cancer was used. Data from the present investigation show for the first time that leptin upregulates the translational and transcriptional expression of all IL-1 system components. Leptin activates several canonical and non-canonical signalling pathways in 4T1 cells mainly leading to increased levels of two transcription factors, SP1 and NF-*κ*B that were involved in IL-1 gene regulation. In addition, leptin-induced activation of PI-3K signalling pathway was related to increased levels of pmTOR, p70S6K1 and p4E-BP. Leptin upregulation of VEGF/VEGFR2 was partially dependant of IL-1 signalling (Figure 8). Moreover, leptin upregulated its own receptor, Ob-R, that could reinforce leptin actions on the expression of IL-1 system. These data strongly suggest that leptin pro-inflammatory and pro-angiogenic effects are closely connected.

Immunosuppression provoked by excessive inflammatory response could lead to increased tumourigenesis and tumour-cell invasion (Voronov *et al*, 2003). Several studies suggest that the IL-1 family of cytokines may be important in regulating protumourigenic activities within the breast cancer microenvironment (Miller *et al*, 2000; Pantschenko *et al*, 2003; Singer *et al*, 2003). IL-1*β* activates the NF-*κ*B pathway and induces both cell migration and proliferation (Wang *et al*, 2005; Lewis *et al*, 2006; Streicher *et al*, 2007). Increased IL-1*α* expression correlated with the expression of prometastatic (*IL-6* and *IL-8*) and anti-apoptotic genes (*TRAF-1* and *cIAP-2*) (Bhat-Nakshatri *et al*, 1998; Newton *et al*, 1999; Patel *et al*, 2000) and is associated with invasive and metastatic cancer leading to poor prognosis (Miller *et al*, 2000). Moreover, increased levels of IL-1, due to functional polymorphism of IL-1*α* gene, enhance hepatocellular carcino susceptibility (Gao *et al*, 2009). Data from IL-1 KO mice strongly suggest that IL-1 is a crucial factor in determining the balance between immunity and inflammation in tumours (Voronov *et al*, 2010).

Tumour growth stimulation by IL-1 system appears to depend on cancer cell–stromal cell interactions. Elevated production and levels of IL-1 system components are found in invasive breast cancer (Apte and Voronov, 2002). However, no studies have been performed on the leptin regulation of IL-1 in breast cancer. Furthermore, it is a lack of information on whether high levels of leptin in obese patients parallel the levels of IL-1 in breast cancer. Indeed, one of the factors secreted by tumour and adipose cells is leptin, a small and pleiotropic cytokine, which could be involved in the regulation of tumour-related inflammatory response. Accumulating evidence suggest that leptin is an important player in breast cancer growth and tumour angiogenesis (Vona-Davis and Rose, 2009; Ray and Cleary, 2010). Elevated levels of leptin/Ob-R in breast cancer are commonly linked to more invasive tumours and worse prognosis (Cleveland *et al*, 2010). Both leptin and Ob-R are over-expressed in cancer tissue relative to non-cancer epithelium (Ishikawa *et al*, 2004). Therefore, our present results open the possibilities to further explore these hypotheses.

Present results show that leptin induced the expression of the Ob-Rb with full-signalling capabilities. Therefore, it is possible that high levels of leptin found in the tumour microenvironment, derived either from tumour or adipose cells, can upregulate the expression of Ob-R by tumour cells leading to the increase of tumour growth (Otvos *et al*, 2008) and expression of pro-angiogenic and inflammatory factors (Guagnano *et al*, 2003). In addition, leptin activates several genes involved in cell proliferation by upregulation of CDK2 and cyclin D1 levels (Dieudonne *et al*, 2002; Okumura *et al*, 2002).

Leptin at concentrations similar to those found in the serum of normal-weight individuals (i.e., 10–20 ng ml^−1^) induced expression of IL-1 system. Therefore, it is anticipated that higher concentrations of leptin as those found in serum from obese individuals could impact on the expression of IL-1 in breast cancer. Leptin-induced expression of IL-1R tI involved several leptin-induced signalling pathways. However, JAK2 and ERK1/2 were mainly involved in the upregulation of IL-1 ligands and antagonist. These findings are in agreement with leptin's ability to upregulate Ob-Rb in 4T1 cells. Ob-Rb is the only leptin receptor that can signal through pSTAT3 (Frühbeck, 2006). In addition, leptin-induced activation of PKC and JNK signalling pathways increased IL-1Ra levels. Therefore, it could be expected that biological processes leading to reduction of PKC (i.e., by activated *ras* oncogene) (Weyman *et al*, 1988) or JNK (i.e., by NF-*κ*B pathway) (Beevers *et al*, 2006; De Smaele *et al*, 2001) could downregulate IL-1Ra and increase the actions of IL-1 agonists. Present findings show that leptin-activated SP1 and NF-*κ*B were involved in the regulation of IL-1*α* promoter

Leptin-mediated activation of mTOR, a pathway deregulated in many cancers (Beevers *et al*, 2006; Knight *et al*, 2006), was involved in the regulation of IL-1 system. Indeed, leptin activation of pAKT1/mTOR/p4E-BP1 increased the levels of IL-1*β*, IL-1Ra and IL-1R tI in 4T1 cells. Present data suggest that IL-1*α* promoter is under regulatory effects of leptin through activated SP1 and NF-*κ*B. It is known that SP1 activation augments the angiogenic and metastatic capacity of tumour cells through overexpression of multiple downstream genes, including *VEGF* (Shi *et al*, 2001). Currently, how leptin activates SP1 in breast cancer cells is unknown.

Because both leptin and IL-1 are inflammatory and pro-angiogeneic factors that upregulate VEGF, we hypothesised that the association between IL-1 and leptin could be a critical event for tumour angiogenesis. Consequently, the blockade of IL-1R tI partially abrogated leptin-mediated increase of both VEGF and VEGFR2 protein and mRNA. These data strongly suggest that leptin pro-angiogenic signature in breast cancer could partially be mediated by IL-1 signalling. These cytokines could actively crosstalk in breast cancer eliciting pro-inflammatory and pro-angiogenic effects that contribute to cancer growth.

## Conclusions

Aberrant levels of inflammatory cytokines are a hallmark of breast cancer. Present study further supports the idea that leptin has an important role in breast cancer by increasing the expression of pro-angiogenic and pro-inflammatory factors. Leptin upregulation of IL-1 system could further enhance leptin's actions in breast cancer. Moreover, leptin and IL-1 could have synergistic functions in breast cancer progression promoting VEGF/VEGFR2 expression (Gonzalez *et al*, 2006; Rene Gonzalez *et al*, 2009; Gonzalez-Perez *et al*, 2010) and tumour macrophage recruitment (Cao *et al*, 2001) that could indirectly augment leptin-mediated angiogenic effects and breast cancer growth. In addition, VEGF signalling transduction is also required to IL-1 induction (Mills *et al*, 2008). Therefore, understanding of how these various factors cooperate to promote tumour progression could lead to the development of more effective combination therapies to fight breast cancer.

## References

[bib1] Apte RN, Dotan S, Elkabets M, White MR, Reich E, Carmi Y, Song C, Dvozkin T, Krelin Y, Voronov E (2006a) The involvement of IL-1 in tumorigenesis, tumor invasiveness, metastasis and tumor-host interactions. Cancer Metastasis Rev 25: 387–408 17043764 10.1007/s10555-006-9004-4

[bib2] Apte RN, Krelin Y, Song X, Dotan S, Rechi E, EIkabets M, Carmi Y, Dvorkin T, White RM, Gayvoronsky L, Segal S, Voronov E (2006b) Effects of micro-environment- and malignant cell-derived interleukin-1 in carcinogenesis, tumour invasiveness and tumour-host interactions. Eur J Cancer 42: 751–759 16530403 10.1016/j.ejca.2006.01.010

[bib3] Apte RN, Voronov E (2002) Interleukin-1-a major pleiotropic cytokine in tumor-host interactions. Semin Cancer Biol 12: 277–290 12147202 10.1016/s1044-579x(02)00014-7

[bib4] Beevers CS, Li F, Liu L, Huang S (2006) Curcumin inhibits the mammalian target of rapamycin-mediated signaling pathways in cancer cells. Int J Cancer 119: 757–764 16550606 10.1002/ijc.21932

[bib5] Bhat-Nakshatri P, Newton TR, Goulet Jr R, Nakshatri H (1998) NF-kappaB activation and interleukin 6 production in fibroblasts by estrogen receptor-negative breast cancer cell-derived interleukin 1alpha. Proc Natl Acad Sci USA 95: 6971–6976 9618523 10.1073/pnas.95.12.6971 PMC22705

[bib6] Boraschi D, Bossu P, Macchia G, Ruggiero P, Tagliabue A (1996) Structure-function relationship in the IL-1 family. Front Biosci 1: 270–308 10.2741/a132 9159234

[bib7] Caldefie-Chézet F, Damez M, de Latour M, Konska G, Mishellani F, Fusillier C, Guerry M, Penault-LIorca F, Guillot J, Vasson MP (2005) Leptin: a proliferative factor for breast cancer? Study on human ductal carcinoma. Biochem Biophys Res Commun 334: 737–741 16009333 10.1016/j.bbrc.2005.06.077

[bib8] Cao R, Brakenhielm E, Wahlestedt C, Thyberg J, Cao Y (2001) Leptin induces vascular permeability and synergistically stimulates angiogenesis with FGF-2 and VEGF. Proc Natl Acad Sci USA 98: 6390–6395 11344271 10.1073/pnas.101564798 PMC33478

[bib9] Carmi Y, Voronov E, Dotan S (2009) The role of macrophage-derived IL-1 in induction and maintenance of angiogenesis. J Immunol 183: 4705–4714 19752225 10.4049/jimmunol.0901511

[bib10] Carino C, Olawaiye AB, Cherfils S, Serikawa T, Lynch MP, Rueda BR, Gonzalez RR (2008) Leptin regulation of proangiogenic molecules in benign and cancerous endometrial cells. Int J Cancer 123: 2782–2790 18798554 10.1002/ijc.23887 PMC2892183

[bib11] Chala E, Manes C, Iliades H, Skaragkas G, Mouratidou D, Kapantais E (2006) Insulin resistance, growth factors and cytokine levels in overweight women with breast cancer before and after chemotherapy. Hormones (Athens) 5: 137–146 16807226 10.14310/horm.2002.11177

[bib12] Chen H, Yang WW, Wen QT, Xu L, Chen M (2009) TGF-beta-induced fibroblast activation protein expression, fibroblast activation protein expression increases the proliferation, adhesion, and migration of HO-8910PM. Exp Mol Pathol 87: 189–194 19747910 10.1016/j.yexmp.2009.09.001

[bib13] Cleary MP, Juneja SC, Phillips FC, Hu X, Grande JP, Maihle NJ (2004) Leptin receptor-deficient MMTV-TGF-alpha/Lepr (*db*) Lepr (*db*) female mice do not develop oncogene-induced mammary tumors. Exp Biol Med (Maywood) 229: 182–193 14734797 10.1177/153537020422900207

[bib14] Cleary MP, Phillips FC, Getzin SC, Jacobson TL, Jacobson MK, Christensen TA, Juneja SC, Grande JP, Maihle NJ (2003) Genetically obese MMTV-TGF-alpha/Lep(*ob*)Lep(*ob*) female mice do not develop mammary tumors. Breast Cancer Res Treat 77: 205–215 12602920 10.1023/a:1021891825399

[bib15] Cleveland RJ, Gammon MD, Long CM, Gaudet MM, Eng SM, Teitelbaum SL, Neugut AI, Santella RM (2010) Common genetic variations in the LEP and LEPR genes, obesity and breast cancer incidence and survival. Breast Cancer Res Treat 120: 745–752 19697123 10.1007/s10549-009-0503-1 PMC3571680

[bib16] Daling JR, Malone KE, Doody DR, Johnson LG, Gralow JR, Porter PL (2001) Relation of body mass index to tumor markers and survival among young women with invasive ductal breast carcinoma. Cancer 92: 720–729 11550140 10.1002/1097-0142(20010815)92:4<720::aid-cncr1375>3.0.co;2-t

[bib17] De Smaele E, Zazzeroni F, Papa S, Nguyen DU, Jin R, Jones J, Cong R, Franzoso G (2001) Induction of gadd45beta by NF-kappaB downregulates pro-apoptotic JNK signalling. Nature 412: 308–313 11713530 10.1038/35104560

[bib18] Dieudonne MN, Machinal-Quelin F, Serazin-Leroy V, Leneveu MC, Pecquery R, Giudicelli Y (2002) Leptin mediates a proliferative response in human MCF7 breast cancer cells. Biochem Biophys Res Commun 293: 622–628 12054648 10.1016/S0006-291X(02)00205-X

[bib19] Frühbeck G (2006) Intracellular signalling pathways activated by leptin. Biochem J 393: 7–20 16336196 10.1042/BJ20051578 PMC1383660

[bib20] Gao Y, He Y, Ding J, Wu K, Hu B, Liu Y, Wu Y, Guo B, Shen Y, Landi D, Landi S, Zhou Y, Liu H (2009) An insertion/deletion polymorphism at miRNA-122-binding site in the interleukin-1alpha 3′ untranslated region confers risk for hepatocellular carcinoma. Carcinogenesis 30: 2064–2069 19917630 10.1093/carcin/bgp283

[bib21] Gonzalez RR, Chefils S, Escobar M, Yoo JH, Carino C, Styer AK, Sullivan BT, Sakamoto H, Olawaiye A, Serikawa T, Lynch MP, Rueda BR (2006) Leptin signaling promotes the growth of mammary tumors and increases the expression of vascular endothelial growth factor (VEGF) and its receptor type two (VEGF-R2). J Biol Chem 281: 26320–26328 16825198 10.1074/jbc.M601991200

[bib22] Gonzalez RR, Leary K, Petrozza JC, Leavis PC (2003) Leptin regulation of the interleukin-1 system in human endometrial cells. Mol Hum Reprod 9: 151–158 12606591 10.1093/molehr/gag022

[bib23] Gonzalez-Perez RR, Xu Y, Guo S, Watters A, Zhou W, Leibovich SJ (2010) Leptin upregulates VEGF in breast cancer via canonic and non-canonic signaling pathways and NFkappaB/HIF-1alpha activation. Cell Signal 22: 1350–1362 20466060 10.1016/j.cellsig.2010.05.003 PMC2928711

[bib24] Guagnano MT, Romano M, Falco A, Nutini M, Marinopiccoli M, Manigrasso MR, Basili S, Davi G (2003) Leptin increase is associated with markers of the hemostatic system in obese healthy women. J Thromb Haemost 1: 2330–2334 14629465 10.1046/j.1538-7836.2003.00445.x

[bib25] Guo S, Colbert L, Fuller M, Zhang Y, Gonzalez-Perez RR (2010) Vascular Endothelial Growth Factor Receptor-2 in Breast Cancer. Biochim Biophys Acta 1806: 108–121 20462514 10.1016/j.bbcan.2010.04.004 PMC2885515

[bib26] Ishikawa M, Kitayama J, Nagawa H (2004) Enhanced expression of leptin and leptin receptor (OB-R) in human breast cancer. Clin Cancer Res 10: 4325–4331 15240518 10.1158/1078-0432.CCR-03-0749

[bib27] Jarde T, Perrier S, Vasson MP, Caldefie-Chezet F (2010) Molecular mechanisms of leptin and adiponectin in breast cancer. Eur J Cancer, Published online 4 October 2010 10.1016/j.ejca.2010.09.005 20889333

[bib28] Johnston A, Arnadottir S, Gudjonsson JE, Aphale A, Sigmarsdottir AA, Gunnarsson SI, Steinsson JT, Elder JT, Valdimarsson H (2008) Obesity in psoriasis: leptin and resistin as mediators of cutaneous inflammation. Br J Dermatol 159: 342–350 18547319 10.1111/j.1365-2133.2008.08655.x PMC2757771

[bib29] Knight ZA, Gonzalez B, Feldman ME, Zunder ER, Goldenberg DD, Williams O, Loewith R, Stokoe D, Balla A, Toth B, Balla T, Weiss WA, Williams RL, Shokat KM (2006) A pharmacological map of the PI3-K family defines a role for p110alpha in insulin signaling. Cell 125: 733–747 16647110 10.1016/j.cell.2006.03.035 PMC2946820

[bib30] Kumar S, Kishimoto H, Chua HL, Badve S, Miller KD, Bigsby RM, Nakshatri H (2003) Interleukin-1 alpha promotes tumor growth and cachexia in MCF-7 xenograft model of breast cancer. Am J Pathol 163: 2531–2541 14633625 10.1016/s0002-9440(10)63608-5 PMC1892398

[bib31] Lewis AM, Varghese S, Xu H, Alexander HR (2006) Interleukin-1 and cancer progression: the emerging role of interleukin-1 receptor antagonist as a novel therapeutic agent in cancer treatment. J Transl Med 4: 485–510 10.1186/1479-5876-4-48 PMC1660548 17096856

[bib32] Mills PJ, Ancoli-Israel S, Parker B, Natarajan L, Hong S, Jain S, Sadler GR, von Känel R (2008) Predictors of inflammation in response to anthracycline-based chemotherapy for breast cancer. Brain Behav Immun 22: 98–104 17706918 10.1016/j.bbi.2007.07.001 PMC2199880

[bib33] Miller LJ, Kurtzman SH, Anderson K, Wang Y, Stankus M, Renna M, Lindquist R, Barrows G, Kreutzer DL (2000) Interleukin-1 family expression in human breast cancer: interleukin-1 receptor antagonist. Cancer Invest 18: 293–302 10808364 10.3109/07357900009012171

[bib34] Miyoshi Y, Funahashi T, Tanaka S, Taguchi T, Tamaki Y, Shimomura I, Noguchi S (2006) High expression of leptin receptor mRNA in breast cancer tissue predicts poor prognosis for patients with high, but not low, serum leptin levels. Int J Cancer 118: 1414–1419 16206269 10.1002/ijc.21543

[bib35] Newton TR, Patel NM, Bhat-Nakshatri P, Stauss CR, Goulet Jr RJ, Nakshatri H (1999) Negative regulation of transactivation function but not DNA binding of NF-kappaB and AP-1 by IkappaBbeta1 in breast cancer cells. J Biol Chem 274: 18827–18835 10373501 10.1074/jbc.274.26.18827

[bib36] Okumura M, Yamamoto M, Sakuma H, Kojima T, Maruyama T, Jamali M, Cooper DR, Yasuda K (2002) Leptin and high glucose stimulate cell proliferation in MCF-7 human breast cancer cells: Reciprocal involvement of PKC-alpha and PPAR expression. Biochim Biophys Acta 1592: 107–116 12379472 10.1016/s0167-4889(02)00276-8

[bib37] Otvos Jr L, Terrasi M, Cascio S, Cassone M, Abbadessa G, De Pascali F, Scolaro L, Knappe D, Stawikowski M, Cudic P, Wade JD, Hoffmann R, Surmacz E (2008) Development of a pharmacologically improved peptide agonist of the leptin receptor. Biochim Biophys Acta 1783: 1745–1754 18555805 10.1016/j.bbamcr.2008.05.007

[bib38] Pantschenko AG, Pushkar I, Anderson KH, Wang Y, Miller LJ, Kurtzman LJ, Kurtzman SH, Barrows G, Kreutzer DL (2003) The interleukin-1 family of cytokines and receptors in human breast cancer: implications for tumour progression. Int J Oncol 23: 269–284 12851675

[bib39] Patel NM, Nozaki S, Shortle NH, Bhat-Nakshatri P, Newton TR, Rice S, Gelfanov V, Boswell SH, Goulet Jr BJ, Sledge Jr GW, Nakshatri H (2000) Paclitaxel sensitivity of breast cancer cells with constitutively active NF-kappaB is enhanced by IkappaBalpha super-repressor and parthenolide. Oncogene 19: 4159–4169 10962577 10.1038/sj.onc.1203768

[bib40] Ray A, Cleary MP (2010) Leptin as a potential therapeutic target for breast cancer prevention and treatment. Expert Opin Ther Targets 14: 443–451 20230196 10.1517/14728221003716466

[bib41] Rene Gonzalez R, Watters A, Xu Y, Singh UP, Mann DR, Rueda BR, Penichet ML (2009) Leptin-signaling inhibition results in efficient anti-tumor activity in estrogen receptor positive or negative breast cancer. Breast Cancer Res 11(3): R36 19531256 10.1186/bcr2321 PMC2716504

[bib42] Shi Q, Le X, Abbruzzese JL, Peng Z, Qian CN, Tang H, Xiong Q, Wang B, Li XC, Xie K (2001) Constitutive SP1 activity is essential for differential constitutive expression of vascular endothelial growth factor in human pancreatic adenocarcinoma. Cancer Res 61: 4143–4154 11358838

[bib43] Singer CF, Kronsteiner N, Hudelist G, Martin E, Walter I, Kubista M, Czerwenka K, Schreiber M, Seifert M, Kubista E (2003) Interleukin 1 system and sex steroid receptor expression in human breast cancer: interleukin 1 alpha protein secretion is correlated with malignant phenotype. Clin Cancer Res 9: 4877–4883 14581361

[bib44] Streicher KL, Willmarth NE, Garcia J, Boerner JL, Dewey TG, Ethier SP (2007) Activation of a nuclear factor kappaB/interleukin-1 positive feedback loop by amphiregulin in human breast cancer cells. Mol Cancer Res 5: 847–861 17670913 10.1158/1541-7786.MCR-06-0427

[bib45] Styer AK, Sullivan BT, Puder M, Arsenault D, Petrozza JC, Serikawa T, Chang S, Hasan T, Gonzalez RR, Rueda BR (2008) Ablation of leptin signaling disrupts the establishment, development, and maintenance of endometriosis-like lesions in a murine model. Endocrinology 149: 506–514 17962343 10.1210/en.2007-1225 PMC2219296

[bib46] Valdivia-Silva JE, Franco-Barraza J, Silva AL, Pont GD, Soldevila G, Meza I, Garcia-Zepeda EA (2009) Effect of pro-inflammatory cytokine stimulation on human breast cancer: implications of chemokine receptor expression in cancer metastasis. Cancer Lett 283: 176–185 19409696 10.1016/j.canlet.2009.03.040

[bib47] Vona-Davis L, Rose DP (2009) Angiogenesis, adipokines and breast cancer. Cytokine Growth Factor Rev 20: 193–201 19520599 10.1016/j.cytogfr.2009.05.007

[bib48] Voronov E, Reich E, Dotan S, Dransh P, Cohen I, Huszar M, Fogel M, Kleinman HK, White RM, Apte RN (2010) Effects of IL-1 molecules on growth patterns of 3-MCA-induced cell lines: an interplay between immunogenicity and invasive potential. J Immunotoxicol 7: 27–38 20001788 10.3109/15476910903405528

[bib49] Voronov E, Shouval DS, Krelin Y, Cagnano E, Benharroch D, Iwakura Y, Dinarello CA, Apet RN (2003) IL-1 is required for tumor invasiveness and angiogenesis. Proc Natl Acad Sci USA 100: 2645–2650 12598651 10.1073/pnas.0437939100 PMC151394

[bib50] Wang FM, Liu HQ, Liu SR, Tang SP, Yang L, Feng GS (2005) SHP-2 promoting migration and metastasis of MCF-7 with loss of E-cadherin, dephosphorylation of FAK and secretion of MMP-9 induced by IL-1beta *in vivo* and *in vitro*. Breast Cancer Res Treat 89: 5–14 15666191 10.1007/s10549-004-1002-z

[bib51] Weyman CM, Taparowsky EJ, Wolfson M, Ashendel CL (1988) Partial down-regulation of protein kinase C in C3H 10T 1/2 mouse fibroblasts transfected with the human Ha-ras oncogene. Cancer Res 48: 6535–6541 3052806

[bib52] Whiteman MK, Hillis SD, Curtis KM, McDonald JA, Wingo PA, Marchbanks PA (2005) Body mass and mortality after breast cancer diagnosis. Cancer Epidemiol Biomarkers Prev 14: 2009–2014 16103453 10.1158/1055-9965.EPI-05-0106

